# Fatty Acid Composition of Dried Fruits of *Sclerocarya birrea*, *Diospyros blancoi* and *Landolphia kirkii*

**DOI:** 10.3390/ijerph14111401

**Published:** 2017-11-17

**Authors:** Athanasia O. Matemu, Durotoye Adeyemi, Hlengilizwe Nyoni, Ladislaus Mdee, Papiso Tshabalala, Bhekie Mamba, Titus A. M. Msagati

**Affiliations:** 1 Nanotechnology and Water Sustainability, College of Science Engineering and Technology, University of South Africa, Johannesburg 1710, South Africa; athanasia.matemu@nm-aist.ac.tz (A.O.M.); dadeyemi@gmail.com (D.A.); nyonih@unisa.ac.za (H.N.); Mambabb@unisa.ac.za (B.M.); 2 Department of Food and Nutritional Sciences, School of Life Sciences and Bioengineering, Nelson Mandela African Institute of Science and Technology, P.O. Box 447 Arusha, Tanzania; 3 School of Medicine, University of Limpopo C/O R71 Tzaneen Road and University Street Mankweng Township, Polokwane University of Limpopo Old Admin Block, Ground Floor Sovenga, Limpopo 0727, South Africa; Ladislaus.Mdee@ul.ac.za; 4 Consumer Goods Council of South Africa, Johannesburg 1710, South Africa; papisot@cgcsa.ac.za

**Keywords:** *Sclerocarya birrea*, *Diospyros blancoi*, *Landolphia kirkii*, GC-TOF-MS, fatty acids

## Abstract

Wild fruits are commonly consumed in the rural communities of South Africa. The information on their nutritionally important fatty acids is, however, limited. Three wild fruit species, *Diospyros blancoi*, *Landolphia kirkii* and *Sclerocarya birrea* from Limpopo Province were selected for evaluation of fatty acid content. Fatty acids composition of dried fruits of *Diospyros blancoi* (Db), *Landolphia kirkii* (Lk) and ripe and/or overripe *Sclerocarya birrea* (Sb) were evaluated by a gas chromatography-time of flight-mass spectrometer (GC-TOF-MS). Hexadecanoic acid (C16:0) was found in highest abundance in *L. kirkii* (57.73–73.55%), followed by *S. birrea* (55.92–71.31%) and *D. blancoi* (46.31–62.05%), respectively. Octadecanoic acid (C18:0) was of second highest abundance, with 24.71–100% in *D. blancoi*, *L. kirkii* (31.03–41.60%) and *S. birrea* (9.11–17.0%). The 9-octadecenoic acid (C18:1*n*-9) was the major unsaturated fatty acid in both *S. birrea* (5.33–18.82%), *D. blancoi* (8.22–8.92%), and *L. kirkii* (3.84–8.63%). The 9,-12-octadecadienoic acid (C18:2*n*-6) was the major unsaturated fatty acid in *D. blancoi* (22.34%). The 9,-12,-15-octadecatrienoic acid (C18:3*n*-3) was found in *L. kirkii* (3.51%) and *S. birrea* (2.79%). From the results, saturated fatty acids were the most dominant, whereas mono- and poly-unsaturated fatty acids were the minor constituents. Therefore, presence of nutritionally important essential fatty acids from *S. birrea*, *D. blancoi* and *L. kirkii* has been shown.

## 1. Introduction

Fruits are an important component of a healthy diet, and their consumption could help prevent a wide range of lifestyle diseases [1]. Wild fruits play an important role in complementing or supplementation of diets to rural populations, due to their nutritional value. Wild fruits contain essential nutrients, however, information on the nutrition composition is limited and fragmented [2]. Wild fruits are healthy types of foods which are known to be rich in minerals, essential fatty acids, vitamins, fiber, bioactive compounds, as well as low in calories. According to [3], bioactive compounds in wild fruits include ascorbic acid (vitamin C), tocopherols (Vitamin E) and polyunsaturated fatty acids.

*Sclerocarya birrea*, *Diospyros blancoi*, and *Landolphia kirkii* are wild fruits geographically distributed in different parts of the world. They are widely consumed as source of nutrients in human diets, mostly in rural settings. The consumption of wild fruits is currently gaining an increasing interest, and it has been associated with an increased intake of a more varied diet contributing to the health and wellbeing of the rural communities [2]. In some societies, wild fruit consumption has become an important component of the dietary traditions [3]. Essential fatty acids are necessary for the cells and good health, and they have to be consumed via diet, as the human body is not capable of producing them [4]. Due to the changing lifestyles and higher consumer demands for specific health foods, more research into functional foods is needed, especially those of natural origin. Demand for plant essential oils has risen as a consequence of consumers searching for cheaper, more “natural” alternatives to disease-fighting medications [4]. Fatty acids constitute the key lipid units, and are required in human nutrition as a source of energy, and for physiological and structural functions. Dietary fats provide essential fatty acids and facilitate the assimilation of fat-soluble vitamins, with saturated, mono- and polyunsaturated fatty acids being the most common [5]. Earlier studies have suggested positive roles played by fruits in the interruption of oxidative stress, ultimately disease prevention, and this effect has been associated with the presence of unsaturated fatty acids, fibers, minerals, and vitamins [6]. Physiological measures of essential fatty acids include promoting health and lowering or preventing the risks of non-communicable diseases. Diets rich in a specific fatty acid may provide potential prevention of a number of health problems or diseases [5].

It is well known that *S. birrea* is a rich source of essential fatty acids, such as 9-octadecenoic and 9,-12-octadecadienoic acids, in addition to other saturated fatty acids [7]. Marula (*S. birrea*) kernel oil was reported to contain nine fatty acids of which hexadecanoic, octadecanoic, and arachidonic acids were the most dominant [8]. The edible pit of *S. birrea* was reported to contain 9-octadecenoic and 9,-12-octadecadienoic acids as essential fatty acids, moreover, 9,-12,-15-octadecatrienoic acid was absent [9]. Fatty acid composition of *S. birrea* has also been reported [10,11], moreover, very little is known about the fruit skin and pulp.

Previous studies have documented fatty acid composition of wild fruit oils from different localities of the world [10,12,13,14,15,16], whereas very little is known about *D. blancoi* and *L. kirkii*. Fatty acid profile of *D. mespiliformis* (*Diospyros* sp.) seed oil was found to contain saturated (39.54%), monounsaturated (29.42%), and poly-unsaturated (29.66%) fatty acids [17]. Considering the importance of these wild fruits in human diet, this study aimed at evaluating and characterizing fatty acid composition of wild dried fruits of *L. kirkii*, *D. blancoi* and *S. birrea* using a state-of-the-art GC-TOF-MS.

## 2. Materials and Methods

### 2.1. Sampling and Storage

*Sclerocarya birrea* (ripe and overripe), *D. blancoi* and *L. kirkii* fruits where collected from Limpopo Province, South Africa in April 2014. A convenience sampling of the wild fruits was done only once, as prescribed by [18], based on the availability of the fruits in the area. Collected fruits were sorted, and approximately 1.5 kg of each fruit category was taken and stored separately in plastic bags, cooled to 4 °C, and immediately transported to the laboratory for further preparations.

### 2.2. Chemicals and Reagents

Methanol, ether and *n*-hexane (HPLC grade) were obtained from *Merck Millipore*, Johannesburg, South Africa. Chloroform, acetone, and ethyl sulfate were obtained from *PAL Chemicals*, Johannesburg, South Africa and sodium sulfate (Na_2_SO_4_) from *Promak Chemicals*, Johannesburg, South Africa. Deionized water (18.4 Micro-Ohms) was prepared using a reverse osmosis water purification system. All other chemicals and reagents used in this study were of analytical grade.

### 2.3. Preparation of Fruits

The collected wild fruits were washed with normal tap water and rinsed with deionized water, then separated into its parts (flesh, pulp, and seeds), based on the type of fruit, and dried at room temperature for 10 days under an extractor, to a final moisture content of below 10%. The dried fruit parts were finely ground and stored at −20 °C for further analysis.

### 2.4. Fatty Acid Compositions

#### 2.4.1. Extraction of Oils

The oils from dried wild fruit powder were extracted by the ultrasonic solid–liquid extraction method of Jerman et al. [19], with some modifications. About 2 g were placed in a wide-mouth reagent bottle with screw caps, followed by addition of 20 mL of acetone/methanol (40:60 *v*/*v*) and incubated in water bath at 60 °C. The mixture was then incubated in an ultrasonicated water bath (Elmasonic S375EL, Elma Schmidbauer GmbH, Singen, Germany) at 60 °C for 90 min. The crude oil extracts were then filtered while hot, using glass-fiber filters (diameter, 47 mm) (Sigma Aldrich, Johannesburg, South Africa). The crude oil extracts were concentrated in a rotary evaporator (Eyela, Tokyo Rikakikai Co., Ltd., Tokyo, Japan) at 40 °C. The dried oil extracts were filtered using 0.2 µm PTFE disc filter (Amicon^®^ Billerica, MA, USA), then the acetone/methanol mixture was evaporated to dryness using nitrogen before derivatization.

#### 2.4.2. GC-TOF-MS Analysis of Fatty Acid Methyl Ester

The fatty acid methyl esters (FAMEs) were prepared using 3 mol/L methanolic HCl (1:2 *v*/*v*) and heated at 60 °C for 1 h in a water bath (Elmasonic S375EL, Elma Schmidbauer GmbH, Singen, Germany) [20]. The derivatized oil was then cooled at room temperature, and 1 mL of distilled water was then added for phase separation. The lipophilic fraction was then recovered using 1 mL of *n*-hexane, followed by 1 mL of ethyl sulfate through vortex-assisted extraction. The extract was then dried over dehydrated sodium sulfate and ether evaporated, leaving the FAMEs, which were then redissolved in 1 mL *n*-hexane for analysis. Samples were filtered through 0.2 µm PTEF disc filters before injection. FAMEs were analyzed by a Gas Chromatograph (Agilent Technologies, Inc., Wilmington, DE, USA) equipped with a LECO Pegasus 4D Time of Flight Mass Spectrometry (TOF-MS) detector, and an Agilent on-column injection system. The primary oven was equipped with a Restek Rxi^®^-5Sil MS, 30 m, 0.25 mm ID, 0.25 μm, and the secondary oven with a Restek Rxi^®^-17Sil MS, 1 m, 0.25 mm ID, 0.25 µm fused silica capillary columns. Helium carrier gas was maintained at a constant flow of 1.4 mL/min. The injection temperature was set at 300 °C, at a split/splitless ratio of 1:10. The oven temperature was programmed as follows: 50 °C held for 0.5 min; ramped from 50 °C to 290 °C at 25 °C/min, then 320 °C at 5 °C/min. The mass spectrometry conditions were set as follows: transfer line temperature of 290 °C and ionization (electron ionization) at −70 eV with source temperature of 250 °C; stored mass range in a range of 45–600 μm; acquisition rate: 100 spectra/s and detector voltage of 300 eV. The fatty acid methyl ester contents were expressed as percentages of the sum of all of the fatty acids analyzed.

### 2.5. Data Analysis

Fatty acids composition was calculated based on the total fatty acid content of the dried fruits using Microsoft Excel (2010, Microsoft Office, Las Vegas, NV, USA. Identification of fatty acids was achieved by comparison of the retention times with the standards, comparison of mass spectra with those in the MS library (National Institute of Standards and Technology—NIST, Gaithersburg, MA, USA).

## 3. Results

### 3.1. Fatty Acid Composition of D. blancoi, S. birrea and L. kirkii

Fatty acid composition of the wild dried fruits of *S. birrea*, *D. blancoi* and *L. kirkii* determined as their methyl esters are presented in Table 1, Table 2 and Table 3. Mostly, saturated, mono-unsaturated, and poly-unsaturated fatty acids were reported. Hexadecanoic (C16:0) and octadecanoic (C18:0) acids were the major saturated fatty acids. Furthermore, 9-octadecenoic (C18:1*n*-9) and palmitoleic (C16:1*n*-7) were mono-unsaturated fatty acids. Hexadecatrieonic (C16:3*n*-3), 9,-12-octadecadienoic (C18:2*n*-6), 9,-12,-15-octadecatrienoic (C18:3*n*-3), octadecadieonic (C18:2*n*-4), and 11,-14-eicosadienoic (C20:2*n*-6) acids were the poly-unsaturated fatty acids reported. In this study, unsaturated fatty acids were observed as the minor components. Other saturated fatty acids found were nonanoic (C9:0), dodecanoic (C12:0), tridecanoic (C13:0), hexacosanoic (C26:0), and octacosanoic (C28:0) acids. Additionally, other fatty acids, including cyclo fatty acids, were found in trace amounts (Table 1, Table 2 and Table 3).

Hexadecanoic acid was found in the following order: LkS > SbSR > SbPOR > SbPR > DbP > LkP > LkF > SbSOR > DbS. Besides, no hexadecanoic acid was observed in DbF (Table 1, Table 2 and Table 3). Likewise, trend for octadecanoic acid was LkP > LkF > DbP > SbSR > SbSOR > SbPR. Furthermore, DbS, LkS and SbPOR were free of octadecanoic acid (Table 1, Table 2 and Table 3). On the other hand, tetradecanoic acid (C14:0) was only found in SbPOR and LkS, while completely absent in *D. blancoi*. Furthermore, 9-octadecenoic and 9,-12-octadecadienoic acids were the unsaturated fatty acids in DbP and DbS, whereas octadecadieonic acid was found only in DbP and LkS (Table 1). *Sclerocarya birrea* constituted of 9-octadecenoic, 9,-12-octadecadienoic, and 9,-12,-15-octadecatrienoic acids as mono- and poly-unsaturated fatty acids (Table 2). Additionally, hexadecatrieonic, palmitoleic, and eicosadienoic acids were only found in *S. birrea*. On the other hand, 9-octadecenoic acid was also found in LkF and LkS, whereas 9,-12-octadecadienoic acid was only found in LkF and 9,-12,-15-octadecatrienoic acid in LkS (Table 2)*.* Furthermore, palmitoleic and hexadecatrieonic acids were also found in *S. birrea* and octadecadieonic acid in DbP and LkS, respectively (Table 1, Table 2 and Table 3). The unsaturated fatty acids were found in *D. blancoi* and *S. birrea* with 9-octadecenoic acid being the most widely distributed, followed by 9,-12-octadecadienoic acid. From the study, it was shown that most of the mono- and poly-unsaturated fatty acids were found in the seeds, as compared to the flesh and pulp.

### 3.2. Effect of Maturity on Fatty Acid Composition in S. birrea Fruit Parts

**Figure 1:**
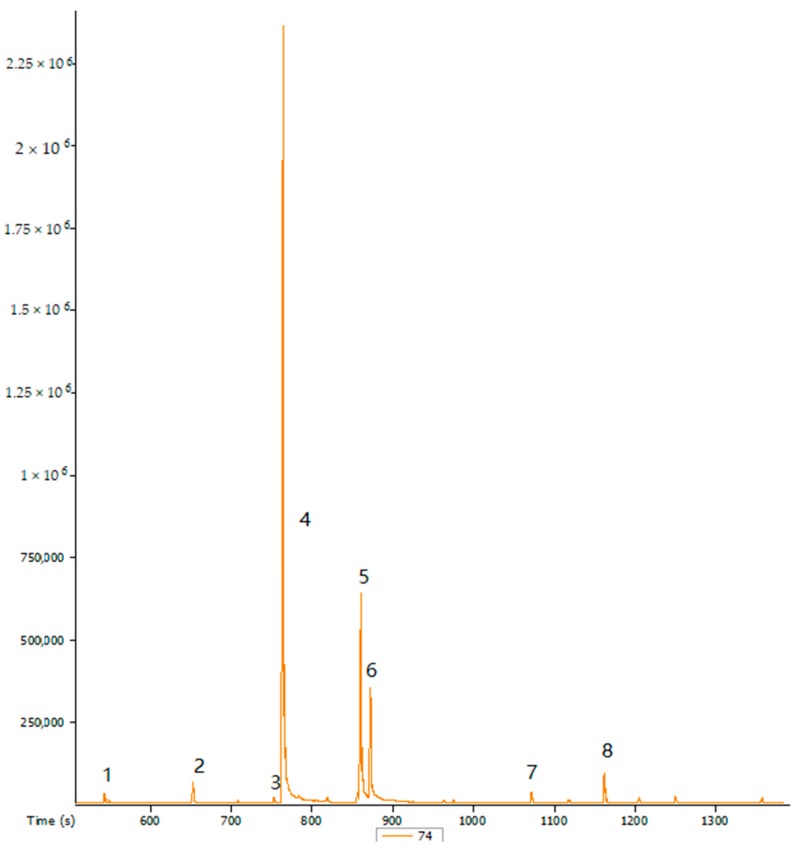
Chromatograms of the fatty acids from the ripe dried pulp of *S. birrea* (SbPR). X-axis represents time (s) and Y-axis represents peak area. 1: Dodecanoic acid, methyl ester; 2: Methyl tetradecanoate; 3: 7-hexadecanoic acid, methyl ester; 4: hexadecanoic acid, methyl ester; 5: 9,-12-octadecadieonic acid, methyl ester; 6: 9,-12-octadecadieonic, methyl ester (Z, Z); 7: Unidentified compound; 8: Tetracosanoic acid. SbPR; Marula (*S. birrea*) ripe dried pulp.

**Figure 2:**
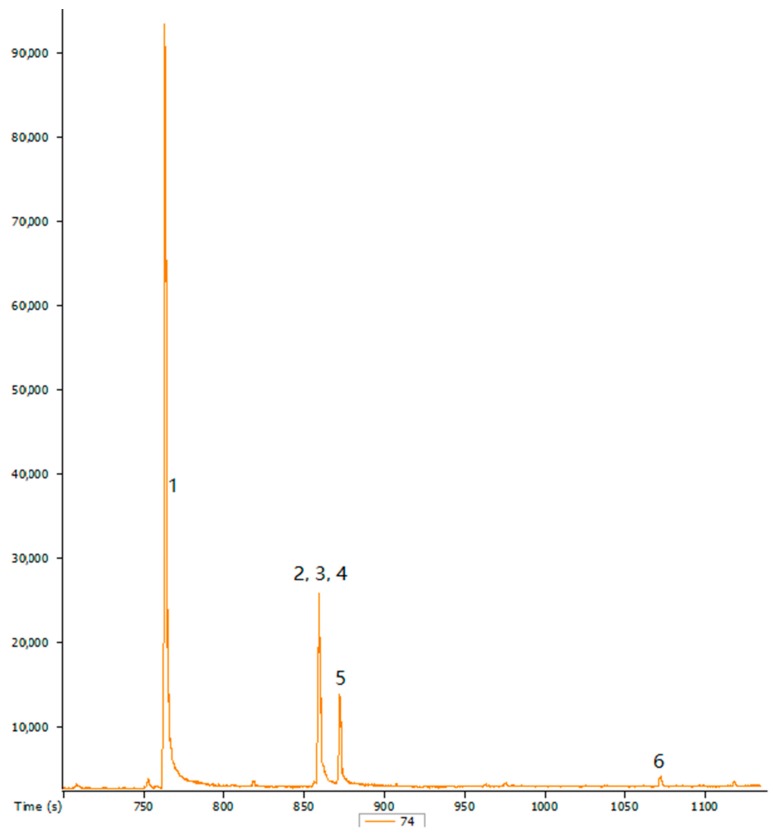
Chromatograms of the fatty acids from the overripe dried pulp of *S. birrea* (SbPOR). X-axis represents time (s) and Y-axis represents peak area. 1: hexadecanoic acid, methyl ester; 2: 9,-12-octadecadenoic acid, methyl ester (E, E); 3: 9-octadecadenoic acid (Z)-, methyl ester; 4: 7,-10,-13-hexadecatrienoic acid, methyl ester; 5: nonanoic acid, methyl ester; 6: hexadecanoic acid, 15-methyl ester. SbPOR; Marula (*S. birrea*) overripe dried pulp.

The effect of maturity on fatty acid composition of oils from *S. birrea* fruit skin and pulp was also evaluated. Different types of fatty acids with their profiles from ripe and overripe *S. birrea* fruit pulp are shown in Figure 1 and Figure 2. Saturated fatty acid, namely hexadecanoic acid, was the major component in both ripe and overripe skin and pulp of *S. birrea*. On the other hand, a declining trend for octadecanoic acid was observed form SbSR, SbSOR to SbPR. Besides, octadecanoic acid was not found in SbPOR. Hexadecanoic (14.16–23%) and octadecanoic (8.84–57%) were the minor acids reported in *S. birrea* kernel [7,8,9], whereas they were the major fatty acids in the skin and pulp of *S. birrea*, as previously reported in Table 2.

The unsaturated fatty acids mainly 9-octadecenoic and 9,-12-octadecadienoic acids were found in both ripe and overripe *S. birrea*. The 9-octadecenoic acid was the major unsaturated fatty acid, followed by 9,-12-octadecadienoic acid. The 9,-12-octadecadienoic acid was found in SbPR and SbPOR as opposed to SbSR (Table 2). Additionally, 9,-12,-15-octadecatrienoic acid was the minor component found only in SbSOR. A similar trend of 9-octadecenoic (4.13–67.25%) and 9,-12,-15-octadecatrienoic acids (4.30–5.93%) from a kernel of *S. birrea* was also reported [8,9].

## 4. Discussion

### 4.1. Fatty Acid Composition of D. blancoi, S. birrea and L. kirkii

Generally, saturated fatty acids, of which hexadecanoic acid was predominant, were observed in all fruits, followed by octadecanoic acid specifically in *D. blancoi* and *L. kirkii* (Table 1, Table 2 and Table 3). Hexadecanoic acid is the most prominent vegetable fat, whereas tetradecanoic and octadecanoic acids form minor components in most vegetable fats [5]. Similarly, 30.06%, 7.74% and 0.64% of hexadecanoic, octadecanoic, and tetradecanoic acids were observed in *D. mespiliformis* seed oil from South Africa [10], a closely related family of *D. blancoi*. Saturated fatty acids were mainly found in flesh, skin, and pulp, with no traces in the seeds. Domination of saturated fatty acids in other fruit oils has also been reported [11,12,13].

Mono- and poly-unsaturated fatty acids were the minor components, among which 9-octadecenoic acid was the main unsaturated fatty acid, followed by 9,-12-octadecadienoic and 9,-12,-15-octadecenoic acids (Table 1, Table 2 and Table 3). From the current study, 9,-12-octadecadienoic acid (22.34%) was the major poly-unsaturated fatty acid found in *D. blancoi* seeds. Similarly, 28.71% of 9,-12-octadecadienoic acid was reported from seeds oils of *D*. *mespiliformis* [10]. Likewise, unsaturated fatty acids as major fatty acids have also been reported in other fruit seed oils [14]. Furthermore, to the best of our knowledge, no studies on the fatty acid composition of *D. blancoi* has been found, therefore, this is the first report on the same.

Fatty acid composition of oils from *S. birrea* has been widely reported with hexadecanoic (12–17.10%), octadecanoic (5.10–11.10%), and 9-octadecenoic (63.19–73.61%) acids found as major fatty acids, followed by 9,-12-octadecadienoic (4.96–8.95%), 9,12,15-octadecatrienoic (0.04–1.10%), and tetradecanoic (trace) acids [8,15,16,17]. Conversely, no studies on the fatty acid compositions of *S. birrea* fruit skin and pulp have been found, therefore, this is the first report on this. Generally, unsaturated fatty acids were reported as minor components in this study, compared to the previous studies of which the main focus was on the fruit kernel. Marula nut is rich in non-drying oil, and contains a large proportion of monounsaturated fatty acids classified as high in 9-octadecenoic acid (70–78%) and natural antioxidants [15,18].

On the other hand, since very little is known about fatty acid composition of *Landolphia kirkii*, findings from this study is the first report on fatty acids composition. *Landolphia kirkii* was found to be rich in Hexadecanoic acid as predominant saturated fatty acid and 9-octadecanoic acid and 9,-12,-octadecadienoic acid as minor components of mono- and poly-unsaturated fatty acids.

It has been reported that nutritionally important mono- and poly-unsaturated fatty acids are 9-octadecenoic, 9,-12-octadecadienoic and 9,-12,-15-octadecatrienoic acids [5]. The 9-octadecenoic acid is found in considerable amounts in both animal and plant sources, whereas 9,-12-octadecadienoic acid occur in all dietary fats, and attain main proportions in most vegetable oils. The 9,-12,-15-octadecatrienoic acid is mainly present in plants, and highly concentrated in some seeds and nuts, as well as in some vegetable oils [5]. According to Simopoulos [19], edible portion of fruits contain only a small amount of mono- and poly-unsaturated fatty acids, as opposed to edible oil seeds. Therefore, mono- and poly-unsaturated fatty acids reported in this study can be of nutritional importance to human health, when consumed as part of their diets.

### 4.2. Effect of Maturity on Fatty Acid Composition in S. birrea

The effect of maturity on fatty acid composition of *S. birrea* fruit parts was also studied. Fruit maturity is associated with fruit quality as a result of ripening or after harvest storage. Maturity determines changes in fatty acid composition in the fruit, in addition to the effect of harvest time on the fruit storage or ripening [20]. From the current study, it has been shown that saturated fatty acids were the major components, whereas unsaturated fatty acids were the minor components in both ripe and overripe *S. birrea.* Furthermore, hexadecanoic, 9-octadecenoic, and 9,-12-octadecadienoic acids were found in all fruit parts, regardless of maturity stage, however, same trend was not observed for other fatty acids (Table 2). Conversely, fatty acid profiles were also found to differ subject to the ripening stage and the fruit part (Figure 1 and Figure 2). Previous studies on the effect of fruit maturity on the fatty acid composition from different species have also been reported [21,22,23]. Therefore, it can be suggested that the variation of fatty acids composition in *S. birrea* may be dependent on the stage of fruit maturity and fruit part analyzed.

Furthermore, variation of fatty acid composition within the fruit parts of the wild species was observed. Generally, sixteen fatty acids were found in *S. birrea*, eight in *L. kirkii*, and six in *D. blancoi*. In addition, presence of other fatty acids (C9:0, C12:0, C13:0, C24:0, C26:0, and C28:0) was found only in some fruit parts (Table 1, Table 2 and Table 3). Similarly, high variability in nutrient contents among naturally occurring populations of the same species has also been reported [24]. Fatty acids consist of essential dietary components necessary for health promotion and prevention of risks of acute and chronic diseases. Adequate consumption of fruits has been associated with significantly lower risk of cardiovascular diseases, diabetes, and some types of cancers [25], in addition, to mental development and improving memories [5]. Poly-unsaturated fatty acids, in particular, 9,-12-octadecadienoic acid are essential for growth and development, with a major role in the prevention and treatment of coronary heart disease, hypertension, diabetes, arthritis, other inflammatory and autoimmune disorders as well as cancer [19]. Fatty acid compositions reported in this study are nutritionally and functionally important for human health benefits. Fatty acid compositions of oils from wild fruits have also been reported [3,12,26,27]. Therefore, the current study gives the first report on the fatty acid compositions of *S. birrea*, *D. blancoi* and *L. kirkii* found in the Limpopo Province. The studied wild fruits can be considered as a source of essential fatty acids.

## 5. Conclusions

In summary, fatty acid compositions of *D. blancoi*, *L. kirkii* and *S. birrea* (skin and pulp) are reported in this study for the first time. Saturated fatty acids were mainly observed, whereas mono- and poly-unsaturated fatty acids were the minor constituents. Fruit skin, flesh, and pulp were the main sources of saturated fatty acids, whereas pulp and seeds were the sources of mono- and polyunsaturated fatty acids. *Sclerocarya birrea* was the major source of 9-octadecenoic acid, while 9,-12-octadecadienoic acid was found in *D. blancoi*. The 9,-12-octadecadienoic acid was found in *L. kirkii* and *S. birrea*. The essential fatty acids reported in this study are of nutritional and physiological importance to human health.

## Figures and Tables

**Table 1:** Fatty acid composition of *D. blancoi* fruit (% total fatty acids) as determined by the Gas Chromatography-Time of Flight-Mass Spectrometer (GC-TOF-MS).

Fatty Acid	DbF	DbP	DbS
Nonanoic acid (C9:0)	-	-	23.13
Hexadecanoic acid (C16:0)	-	62.05	46.31
Octadecanoic acid (C18:0)	100	24.71	-
9-Octadecenoic (C18:1*n*-9)	-	8.92	8.22
9,12-Octadecadienoic (C18:2*n*-6)	-	-	22.34
Octadecadieonic acid (C18:2*n*-4)	-	4.32	-
Total	100	100	100

-: not found. *D. blancoi* Seed, DbS; *D. blancoi* Pulp, DbP; *D. blancoi* Flesh, DbF.

**Table 2:** Fatty acid composition of *S. birrea* fruit skin and pulp (% total fatty acids) as determined by the Gas Chromatography-Time of Flight-Mass Spectrometer (GC-TOF-MS).

Fatty Acid	SbSR	SbSOR	SbPR	SbPOR
Nonanoic acid (C9:0)	0.29	2.45	-	7.14
Dodecanoic acid (C12:0)	-	-	0.27	-
Tridecanoic acid (C13:0)	-	5.12	-	-
Tetradecanoic acid (C14:0)	-	-	2.16	-
Hexadecanoic acid (C16:0)	71.31	55.92	67.03	68.34
Palmitoleic acid (C16:1*n*-7)	1.23	3.01	0.66	-
7-Hexadecenoic acid (C16:1*n*-9)	-	-	0.66	-
Hexadecatrieonic acid (C16:3*n*-3)	-	-	-	7.70
Octadecanoic acid (C18:0)	17.0	12.70	9.11	-
9-Octadecenoic (C18:1*n*-9)	5.33	12.97	18.82	15.35
9,-12-Octadecadienoic (C18:2*n*-6)	4.85	-	0.24	1.46
9,-12,-15-Octadecatrienoic acid (C18:3*n*-3)	-	2.79	-	-
Eicosadienoic acid (C20:2*n*-6)	-	1.93	-	-
Tetracosanoic acid (24:0)	-	-	1.62	-
Hexacosanoic acid (C26:0)	-	-	0.40	-
Octacosanoic acid (C28:0)	-	-	0.37	-
Cyclo fatty acids	-	0.76	-	-
Total	100	97.65	99.91	99.99

-: not found. *S. birrea* Skin Ripe, SbSR; *S. birrea* Pulp Ripe, SbPR; *S. birrea* skin Overripe, SbSOR; *S. birrea* Pulp Overripe, SbPOR.

**Table 3:** Fatty acid composition of *L. kirkii* fruit (% of total fatty acids) as determined by the Gas Chromatography-Time of Flight-Mass Spectrometer (GC-TOF-MS).

Fatty Acid	LkF	LkP	LkS
Tetradecanoic acid (C14:0)	-	-	6.05
Hexadecanoic acid (C16:0)	57.73	58.40	73.55
Octadecanoic acid (C18:0)	31.03	41.60	-
9-Octadecenoic (C18:1*n*-9)	3.84	-	8.63
9,-12-Octadecadienoic (C18:2*n*-6)	6.23	-	-
9,-12,-15-Octadecatrienoic acid (C18:3*n*-3)	-	-	3.51
Octadecadieonic acid (C18:2*n*-4)	-	-	4.96
Cyclo fatty acids	1.17	-	3.31
Total	100	100	100

-: not found. *L. kirkii* flesh, LkF; *L. kirkii* pulp, LkP; *L. kirkii* seeds, LkS.
